# Seroprevalence of SARS-CoV-2 antibodies in radio and television workers

**DOI:** 10.17179/excli2021-4545

**Published:** 2022-01-12

**Authors:** Paulo Ricardo Martins-Filho, Danilo Nobre da Silva, Danillo Menezes dos Santos, Márcia Santos Rezende, Jessica Paloma Rosa Silva, Josafá Bonifácio da Silva Neto, Dulce Marta Schimieguel, Lucindo José Quintans-Júnior, Jullyana de Souza Siqueira Quintans, Paula Santos Nunes, Adriano Antunes de Souza Araújo

**Affiliations:** 1 Investigative Pathology Laboratory, Federal University of Sergipe, Aracaju, Sergipe, Brazil; 2 Health Sciences Graduate Program, Federal University of Sergipe, Aracaju, Sergipe, Brazil; 3 Graduate Program in Pharmaceutical Sciences, Federal University of Sergipe, São Cristóvão, Sergipe, Brazil; 4 Department of Pharmacy, Federal University of Sergipe, São Cristóvão, Sergipe, Brazil; 5 Graduate Program in Applied Health Sciences, Federal University of Sergipe, Lagarto, Sergipe, Brazil; 6 Journalism Sector, Radio UFS, Federal University of Sergipe, Aracaju, Sergipe, Brazil

**Keywords:** COVID-19, SARS-CoV-2, mass media

## Abstract

We investigated the seroprevalence of SARS-CoV-2 antibodies in individuals working in radio and television stations (TV) in Sergipe state, Northeast Brazil. This cross-sectional study was conducted from December 1 to December 20, 2020, a period which was considered as the beginning of the second wave of COVID-19 in the state. One hundred and thirteen professionals from the three largest media companies in the state were included in this study. Venous blood was collected using venipuncture and a fluorescence immunoassay for qualitative detection and differentiation of IgM and IgG antibodies against SARS-CoV-2 was performed. Twenty-eight media workers had detectable levels of SARS-CoV-2 antibodies (11 IgM+, 6 IgM+/ IgG+, and 11 IgG+) and the estimated seroprevalence was 24.8 % (95 % CI 17.7 - 33.5). Our findings showed a high seroprevalence of SARS-CoV-2 antibodies in radio and TV workers during the second wave of COVID-19 in Brazil.

## ⁯

***Dear Editor,***

The COVID-19 pandemic has presented an unprecedented challenge for journalism (Ferreira and Borges, 2020[6]). Apart from the uncertainty about job security, media workers are at increased risk of SARS-CoV-2 infection due to the daily routine of reporting in the field. The Press Emblem Campaign (PEC) - an international independent nonprofit and non-governmental organization - has reported that at least 1891 journalists have died from COVID-19 in 86 countries since March 2020. Brazil ranks first in the number of deaths, followed by India and Peru (https://pressemblem.ch/-1.shtml).

To the best of our knowledge, no seroepidemiological studies have yet been conducted that analyzed the presence of SARS-CoV-2 antibodies in broadcast media staff working in both radio and television (TV). Surveillance of antibody seropositivity can help to evaluate the extent of infection in a population and the proportion of people that remains susceptible to the virus. In this study, we investigated the seroprevalence of SARS-CoV-2 antibodies in radio and TV workers in Sergipe state, Northeast Brazil. 

This cross-sectional study was conducted from December 1 to December 20, 2020, a period considered as the beginning of the second wave of COVID-19 in the state. Sergipe is the smallest state in Brazil, and is located in the poorest region of the country. The state has an estimated population of ~2.3 million people and a Human Development Index (HDI) of 0.665. The first case of COVID-19 in Sergipe was confirmed on March 14, 2020, and at the time of writing this manuscript more than 278,000 cases and 6000 deaths had been registered. 

Professionals from the three largest media companies in the state were included in this study. After obtaining written informed consent to participate, individuals were interviewed using a structured questionnaire that included demographic and clinical features. Venous blood was collected aseptically using venipuncture and a fluorescence immunoassay (FIA) (iChroma II, BioSys + Kovalent) for qualitative detection and differentiation of IgM and IgG antibodies against SARS-CoV-2 was performed. A result was considered negative if the automated reader obtained a readout of <0.8, indeterminate if ≥0.8 and <1.1, and positive if ≥1.1. The sensitivity and specificity of the FIA are 95.8 % and 97.0 %, respectively, according to the manufacturer when assessed on 46 SARS-CoV-2 positive patients and 131 negative controls (http://www.biosys.com.br/wp-content/themes/transport/covid-19-images/SOLUCAOPOCT-COMPLETA-COVID-19.pdf). 

The main outcome in the present study was seroprevalence expressed as the proportion of individuals who had a positive result in the FIA. Radio and TV workers were grouped according to their occupation activity as following: (1) production team; (2) reporting team; and (3) operational team. This study was approved by the Ethics Committee of the Federal University of Sergipe (protocol number 33095120.4.0000.5546).

The study included a convenience sample of 113 media workers. The median age (interquartile range [IQR]) was 39 years (IQR, 30.0 - 48.0) and the majority were male (n = 79, 70.0 %). The presence of comorbidities (diabetes, hypertension, cardiac disease, or other chronic condition) was reported by 29 (25.7 %) individuals. Forty-two (37.2 %) workers described close contact with people presenting SARS-CoV-2 infection in the previous two weeks of the serological assay, and the use of facial mask during work activities was reported in most cases (n = 110, 97.3 %). Twenty-eight media workers had detectable levels of SARS-CoV-2 antibodies (11 IgM+, 6 IgM+/ IgG+, and 11 IgG+) and the estimated seroprevalence was 24.8 % (95 % CI 17.7 - 33.5). The highest rates were found among workers in the operational team (27.6 %, 95 % CI 14.7 - 45.7), followed by the production team (25.8 %, 95 % CI 16.6 - 37.9) and the reporting team (18.2 %, 95 % CI 7.3 - 38.5) (Table 1(Tab. 1)). 

Despite the massive interest in social networking services for information sharing and Internet use, the traditional media remains the most important channel through which COVID-19 information is communicated (Frissen et al., 2020[7]). Faced with the need for real-time coverage of COVID-19 information in several settings, broadcast media workers are at an increased risk of SARS-CoV-2 infection. Ensuring that media workers have the required personal protective equipment (PPE) and support they need to work safely has become critical during this unprecedented global sanitary crisis. 

In this study, we found high SARS-CoV-2 seroprevalence estimates in radio and TV workers, and a large proportion of individuals with serological results suggestive of active phase or recent infection. Previous studies have shown a seroprevalence for the general population of 9.3 % (95 % CI 8.5 -10.1) during the first wave (de Souza Araújo et al., 2021[4]) and 15.4 % (95 % CI 14.5 - 16.4) during the second wave of COVID-19 in Sergipe state (de Souza Araújo et al., 2021[5]). These findings indicate high exposure of media workers to SARS-CoV-2, and the presence of these individuals in the work environment unaware that they are infected with the virus. There is evidence that SARS-CoV-2 may spread asymptomatically in a population, even under social distancing restrictions (Cloutier et al., 2021[3]). 

Interestingly, our results showed a lower seroprevalence for SARS-CoV-2 antibodies among journalists, reporters, and videographers. It is possible that reporting teams are more careful in respect of COVID-19 protective measures as they are seen as a high-risk group for SARS-CoV-2 infection due to the need for contact with the public during work activities. In contrast, production and operational teams may have an increased risk of infection because they work in an indoor environment in TV newsrooms. There is evidence that closed indoor spaces with minimal ventilation provide an ideal environment for SARS-CoV-2 transmission (Azuma et al., 2020[1]; Chang et al., 2021[2]).

Our findings showed a high seroprevalence of SARS-CoV-2 antibodies in radio and TV workers during the second wave of COVID-19 in Brazil. Prevention and control protocols against COVID-19 should be revised and implemented by media companies. Further seroepidemiological studies should evaluate the exposure of freelancers and print and digital media professionals to SARS-CoV-2 infection. 

## Declaration

### Competing interest statement

The authors have no competing interests to declare.

### Authors' contributions

All authors contributed equally to the manuscript. 

### Acknowledgment and funding

This study is part of the EpiSERGIPE project which is supported by grant SES/FAPESE/UFS 001/2020. The funding source had no role in the design and conduct of the study; collection, management, analysis, and interpretation of the data; preparation, review, or approval of the manuscript; and the decision to submit the manuscript for publication.

## Figures and Tables

**Table 1:**
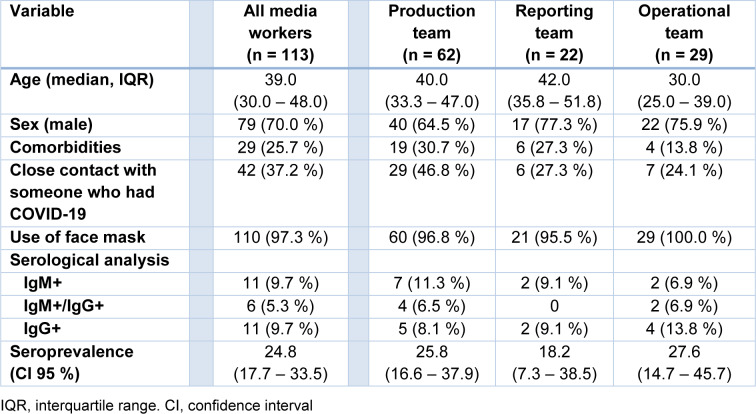
Clinical characteristics and seroprevalence of SARS-CoV-2 antibodies among radio and TV workers
